# Research on the Relationship Between Service Guarantee Perception and Customer Value in the Chinese Context

**DOI:** 10.3389/fpsyg.2021.766098

**Published:** 2022-01-05

**Authors:** Huang-he Yu, Shu-kuan Zhao, Mao-Chou Hsu

**Affiliations:** ^1^School of Management, Jilin University, Changchun, China; ^2^Department of Recreation and Sport Management, Tajen University, Pingtung, Taiwan

**Keywords:** service guarantee perception, customer value, perceived risk, trust of contract, signal theory

## Abstract

As an excellent management tool, service guarantee can improve the competitive advantage of enterprises and allow consumers to obtain high-quality products and services. However, in the current Chinese context, this tool has not played its proper function. One important reason is the perception deviation of Chinese consumers. This research analyzes the main reasons for this deviation, puts forward related hypotheses and research models, and discusses the influence of disposition to trust of contract, perceived structural assurance (PSA), and subjective norm on service guarantee perception (SGP). Also, this study discusses SGP of customers through perceived risk and quality. Through the verification of 574 sample data, the main conclusions are as follows: (1) Disposition to trust of contract, subjective norms, and PSA significantly affect SGP positively; (2) SGP positively affects customer value (CUV); and (3) SGP s are obviously different between people of different ages, education levels, and income levels. Hopefully, these conclusions can have the following enlightenment to enterprises serving Chinese consumers: (1) in the designing stage of service guarantee, perception of customers of this guarantee should be a consideration; (2) CUV can be a proper direction if an enterprise wish to lead the guarantee perception of the customer; (3) Not all products need the same level of service guarantee; and (4)The proper service guarantee level depends on various statistical characteristics of target customers of the service.

## Introduction

The Chinese economy has been in a stage of sustained, rapid, and steady growth for decades. According to official data from the National Bureau of Statistics, since the Chinese government implemented the reform and opening policy in 1978, the economy has developed rapidly. The gross domestic product has risen from 364.5 billion yuan in 1978 to 1,015,589 trillion yuan in 2021, which shows an average annual increase of 9.2% and a volume increase by 279 times. The “disposable income per resident,” as the most intuitive indicator of the national consumption capacity, has increased from 18,311 Yuan since statistics were available in 2013 to 32,189 Yuan in 2020. However, an increase in commercial fraud such as poor service and shoddy goods happens at the same time, especially after the rise of e-commerce (according to the data from China Consumers Association, complaints about online shopping and services account for nearly 40% of the total).

The previous studies have shown that providing quality assurance for services not only prompts companies to focus on customer needs, reduce customer-perceived risks (PERs), and build consumption confidence to achieve the purpose of attracting customers (Kandampully and Butler, 2001) but also helps customers form relatively accurate service expectations, giving them the right to complain, and stipulating service remediation guidelines for employees (Lee, 2006). The practices of some companies in Western countries have also confirmed this, for example, “100% Customer Satisfaction Guarantee System” of Hampton Inn brought in a net income of US$12 million (Sowder, 1996). Eateries used a “special service guarantee” to increase sales by 25% and triple their net profit (Firnstahl, 1989).

However, such an excellent management tool which improves transaction efficiency and service quality, which consequently enables merchants to increase revenue, has received completely different results in the Chinese market. Especially with the rapid development of e-commerce market, in where it is very difficult to supervise the fulfillment of promises. Many service guarantee failures have appeared. It was not uncommon for merchants to refuse to fulfill their promises due to various reasons, such as refusal to return goods or compensation. The government tried to diminish such phenomenon from the legal and regulatory level and designated March 15 as the “Consumer Rights Day” when many typical cases were revealed and publicized. However, the attitude of consumers toward merchants are biased. Consumers will interpret the guarantees of merchants as “possibly unfulfilled” or even “the merchant’s trick to entice them to buy.” It was not good to play the above-mentioned due role, so the research on the service guarantee perception (SGP) of Chinese consumers was particularly important.

As an effective approach of promotion and quality management, service guarantee has attracted great attention from academia. In the late 1980s, scholars began to conduct case studies on companies implementing service guarantees (Evans et al., 1996). It was mainly from the corporate management level to discuss the issue of why service guarantees should be provided and clarified the conceptual framework, basic types, characteristics, and functions. In the mid-to-late 1990s, what service guarantees companies should provide has become the focus of research, and empirical studies on the relationship between service guarantees and customer behaviors began to appear (Tucciand Talaga, 1997). After 2,000, the mechanism of service guarantee (Hays and Hill, 2006), the construction of service guarantee management integration model, and the role of adjustment factors (Marmorstein et al., 2001) have become the main research content. Recently, the service guarantee activation (or abuse) of consumers (Wirtz and Kum, 2004), cross-industry service guarantee research (Wirtz and Kum, 2001), the role of guarantee in the context of service failure and remediation and its influence on post-purchase behavior (Lee, 2006), and other issues began to receive attention. There was still insufficient empirical research on the SGP in the Chinese context.

Therefore, this research will clarify the related concepts of service guarantee and its influence on enterprises and consumers in the Chinese context. Through literature analysis, certain variables would be discussed, including “disposition to trust of contract,” “subjective norms,” “perceived structural assurance,” “service guarantee perception,” “perceived risk,” “perceived quality,” and “customer value.” A research model and relevant hypotheses would be put forward. Through questionnaire surveys and data analysis, this study could have further understanding of the research constructs and verify the hypotheses. The results would clarify whether service guarantees in the Chinese context can increase value customers. It can guide companies to design and use the service guarantees for Chinese consumers.

## Literature Review and Hypotheses Development

### Service Guarantee Perception

Due to the complexity of the concept of “service guarantee,” it has been studied for a long time. However, scholars have not reached a consensus on the precise definition of service guarantee. This concept first appeared in the research by Hart. Hart defined it as a statement of remedy as an enterprise used to explain how the serviced customer can expect the enterprise to perform when the service fails (Hart, 1988). It is a monetary or non-monetary compensation that a customer can require the service provider to pay when the service delivery system does not meet certain standards (Baker and Collier, 2005). A service guarantee was a form of expression of the responsibility attached to the service itself (Boshoff, 2002, 2003). Service guarantee was a measurement standard of service quality and a solution when the standard was not met (Lidén, 2009). The common points of these studies are explained: service guarantee was a kind of information that enterprises convey to consumers through promises, which explains the quality promise (the level of their service quality will reach), and the compensation promise (the compensation standards and procedures).

The purpose of transmitting such kind of information, that is, the role of service guarantee, was mainly concentrated in the following aspects: service guarantee can reduce the PER of customers and the possibility of customer inferred service failure (Tucciand Talaga, 1997), improve the reliability of service quality and the overall evaluation of the company (Marmorstein et al., 2001), enhance customer confidence (Biswas et al., 2006), stimulate customer purchase intentions (Wirtz and Kum, 2001), and induce customer choices (McCollough and Gremler, 2004). A study by Crisafulli and Singh (2016) found that service guarantee can also make consumers feel fair. The above-mentioned effects of service guarantee will be adjusted by factors such as corporate reputation, service quality stability, and customer beliefs in the early stage.

From the above effects, in the industry and academia, “service guarantee” was recognized to attract or retain customers, and it was also a new field in which companies compete (Wirtz and Kum, 2004). If used properly, the service guarantee will bring a huge competitive advantage to the enterprise. However, Anderson and Weitz (1992) pointed out in a 1992 study that if “service guarantee” was used as a dependent variable of a research framework, the perception of each subject involved in the guarantee will be an important parameter. There are three steps in the process of “service guarantee”: the enterprise first makes a guarantee, then consumers form their own subjective cognition after knowing this guarantee, and, finally, consumers make their own behavior according to this cognition. It must be noted that the cognition of people of things can be far from the facts. In this case, consumers can positively understand the guarantee of services by the enterprise as an attempt to create greater customer value (CUV), or negatively understand the guarantee as “it’s simply to stimulate purchase intention the enterprise has planned to use various methods to evade responsibility in after-sales service,” “Enterprises are stimulating customers’ impulsive consumption,” and/or “it’s just a reason of the enterprise’s price inflation, “etc. Therefore, the second step is particularly important. Meaning it can be assumed that “an important antecedent for enterprises to obtain advantages through service guarantee is that consumers have a correct perception of enterprise guarantee.”

### Disposition to Trust of Contract

Generalized trust expresses a fundamental propensity to trust other people that varies between countries based on religious and ethnic composition, inequality, quality of government, and welfare regime. It furthermore varies between individuals based on level of education, health, employment, and compositions of residential area (Frederiksen, 2019). In the process of the understanding of consumers of the service guarantees provided by businesses, the preconceived disposition of consumers to trust contracts play an important role. Just as Mayer et al. (1995) said in their research, when people are establishing social relationships and conducting social activities, it was unavoidable to be affected by the subconsciousness of trust disposition. Trust disposition refers to a general attitude and belief in human nature when people get along with others (McKnight et al., 2002). This disposition was not aimed at a specific organization or individual, but a generalization of the trust of people formed in the process of personal life experience and socialization (McKnight et al., 1998).

The disposition to trust was subjective and varies from person to person. The reason for this difference was that Rotter put forward the attitude of trust in humanity and socialization in 1980 (Rotter, 1980). As early as 1964, Blau (1964) pointed out in his research that cultural background will affect the disposition of everyone to trust in a society. The above consideration of beliefs, culture, and personal values as variables inspired this research. Due to the obvious differences between Chinese and Western societies, a considerable number of Chinese consumers have weak trust in contracts. The promise of service on the shelf was understood as a trap for merchants to induce them to buy or pay high fees. Therefore, the first hypothesis of this study is as follows:

**H1**: The disposition to trust contract will positively affect the SGP of the customer significantly.

### Perceived Structural Assurance

As early as the 1980s, scholars such as Zucker (1986) conducted preliminary studies on the concept of structural assurance, in which he used the authoritative third-party organization, Better Business Bureau as an example of assurance. Then, in a 2002 study on the trust of organizations in the online environment by McKnight et al. (2002), it was summarized as the degree of success of the implementation of the customer of security protection measures such as legal resources, guarantees, commitments, rules, and regulations under certain specific circumstances. A concept was often used as a prerequisite to study trust in organizations (Pavlou and Gefen, 2004) or specific technologies (Ratnasingam and Pavlou, 2003). Structural assurance (SA) positively moderates the trust–continuance intention relationship but not the satisfaction–trust relationship. SA is positively associated with trust (McCole et al., 2019).

The perceived structural assurance (PSA) in this study refers to the perception of consumers of the degree of protection that structural assurance can provide to consumers and their strong willingness to use structural assurance. As this research focuses on the Chinese context, this concept was particularly important. The influence of the Chinese context on structural assurance mainly comes from two aspects. The first is the cultural level, that is, due to the deep-rooted “harmony as the most precious” thought in Chinese culture. The traditional cultural background based on the clan system makes the resolution of contradictions more inclined to the third person (peacemaker). The use of structural assurance to solve problems (appeal to law), nevertheless of the result, will be regarded as a failure at the cultural level (Fei, 2015). The second was the historical reason: the process of marketization (denationalization) of the domestic market of China began in 1978, and the initial development was relatively slow. It was not until 1992 that it began to develop at a high speed. During this period, China completed the transition from a planned economy to a market economy, and the structural assurance related to the market economy have also gone through the process of starting from nothing. So far, it still needs to be improved in many fields (Chen, 1999). This has caused many Chinese consumers to experience a long period of severely imperfect market structural assurance. During this period, many attempts to use structural assurance failed. It becomes a negative view, including distrust and unreliability, very high cost of use, and being inoperable at all. After considering the above two points, we believe that it was necessary to use “perceived structural assurance” as an influencing factor of “perceived service guarantee” in the Chinese context. Therefore, the second hypothesis of this study is:

**H2**: The PSA will positively affect the SGP of the customer significantly.

### Subjective Norms

The concept of subjective norms (SUN) originated from the well-known “Theory of Reasoned Action,” which was proposed by Fishbein and Ajzen (1975). It was a widely used general model to analyze the motivation of the behavior of people. The SUN can be defined as the social pressure that an individual perception about whether to take a behavior. The SUN are the degree of the influence from others on whether an individual takes a behavior (Fishbein and Ajzen, 1975). Existing research has divided SUN into two factors which are directive norms and exemplary norms, both of which reflect the pressure and constraint that social influences exert on individuals, and they have independent effects on behavioral intentions. Directive norms refer to the requirements or expectations of the organization or leadership for employees, which can be either in the form of various written rules and regulations or temporary verbal instructions from leaders, while exemplary norms refer to the influence effect generated by the positive performance of important reference individuals around employees (such as leaders and colleagues with similar seniority; Chatzisarantis and Biddle, 1998).

These two sub-dimensions gave this research a great inspiration because the effect of SUN may vary with time and culture. Recently, several researchers attempted to explain how SUN affect behavioral intentions of individuals to use technology via a cultural perspective (Srite, 2006; Tarhini et al., 2015; Teo et al., 2018; Huang, 2019; Huang et al., 2019). Ordinary consumers in the Chinese context have a high level of motivation to be consistent with the opinions of others. The reason for maintaining this high level was the famous “herd effect” in psychology (Le Bon, 1897), and the “golden mean” in traditional Chinese philosophical thinking. The golden mean has a long history as philosophical thought, and it has two core ideas: (1) behavior should change and match with the surrounding environment; (2) the opposition to extremism (Huang, 2020). Under the influence of this kind of thought, when Chinese people face two completely different views, such as whether they should choose service guarantee, comparing to the Westerners, it was not easy to draw a clear self-judgment (Peng and Nisbett, 1999). Instead, most of them would choose to achieve harmony and unity with others and the surrounding environment without conflict. This conclusion has even been verified by Chinese scholars through the principles of fuzzy mathematics (Liao and Xu, 2012). In this context, the preference of Chinese consumers was more susceptible to the influence of people around them than Western consumers, that is, “exemplary norms.” Therefore, the following assumptions are particularly important, namely:

**H3**: The SUN will positively affect the SGP of customers significantly.

### Customer Value

The above discussion focuses on the relevant factors that affect SGP and the influence of Chinese customers. Service providers offer service promises, hoping that their services can provide customers with more value, thereby enhancing corporate competitiveness. Therefore, it was very necessary to discuss the value of customers.

The earliest theoretical model of CUV was that the difference between the benefits (F) felt by customers purchasing and using products and services and the cost (C). The value the company provides to customers (V), namely F = V-C. Parasuraman et al. (1988) defined the perceived service quality of customers in 1988 as the overall evaluation of the utility of the product or service after weighing the perceived benefits with the cost of acquiring the product or service. Later, Monroe said in his book 2 years later that CUV was not so much the difference between benefits and costs. Mainly, that it is better to consider the ratio of the two, that is, F = V/C (Monroe, 2002). This form of ratio more intuitively expresses that the value of the customer comes from the income in exchange for every penny paid, rather than the logic of offset or deduction like subtraction.

On this basis, Heskett et al. (1994) introduced the quality of the service process and the cost of obtaining services into the calculation of CUV when they studied CUV in the service industry in 1994. They believed that in the process of perceiving value, customers not only pay attention to the products and services but also to the value created by their continuous relationship with the company. Thus, the creation of CUV extends from an “episode” in the transaction to a long-term process, including pre-sales and after-sales, and proposes “Total Episode Value” (TEV) = (core product + core service)/(price + relationship cost). Furthermore, Hogan et al. (2002) put forward their view that CUV can be regarded as a collection of supply value, brand value, and relationship value. It is worth mentioning that CUV is the sum of “core value” and “added value,” and that the added value here may be negative (Ravald and Gronroos, 1996).

It can be clearly seen from the above-mentioned research that academia expands the scope of CUV from products to services and extends the core value obtained from transactions to the value generated by additional services and the value of services. Services have also expanded from positive contributions to the sum of all service utilities, which can be positive or negative. These conclusions are undoubtedly more and more comprehensive and closer to the facts. The research focus of this article, which is “service guarantee,” will increase the value of customers in any calculation. Therefore, we will discuss the SGP as a mediating variable in the Chinese context.

### Perceived Risk

The concept of PER is derived from psychology. Dowling and Staelin (1994) defined PER as the uncertainty and the possibility of adverse consequences that consumers perceive when purchasing products or services. In the process of studying this conceptual system, to establish a research model, scholars divided the PER into many different dimensions. At first, Jacoby et al. divided the PER into psychological risk, financial risk, physical risk, social risk, performance risk, and time risk (Jacoby and Kaplan, 1972). Later, Peter researched this basis and verified that these six dimensions can basically explain more than 90% of the PER (Peter and Ryan, 1976). Based on these dimensions, scholars have established some theoretical models, including a two-factor model, a multi-dimensional model, an inherent risk-operational model, and a comprehensive PER model (Cui, 2019). In short, the dimensions of PER will change as the research field changes. The characteristic of this change mainly stems from the subjectivity of PER. It is different from the objective economic risks discussed in economics. The PER is the subjective feelings of individuals, which leads to the conclusion that the risks that consumers feel subjectively are different from the risks they face objectively. If a certain actual risk is not perceived by consumers, no matter how big the risk is, and what serious consequences will be produced, it will not affect the buying behavior of consumers. Conversely, even if certain risks perceived by consumers do not actually exist, they will affect shopping decisions and behaviors of customers (Mitchell and Boustani, 1993).

This corresponds to the subject of this article, SGP, that is, the service guarantee itself reduces the objective risk of customers (Wu et al., 2012). However, the SGP that exhibits considerable volatility in the Chinese context affects consumption. The nature of the behavior of the user is the main reason why this study sets the PER as a related variable of the SGP. In addition, the perception of risk will be understood as an increase in cost, which will affect the judgment of the final value (Sweeney et al., 1999). We have reason to put forward the following hypothesis:

**H4**: Service guarantee perception will negatively affect the PER of customers significantly.

**H5**: Perceived risk will negatively affect CUV significantly.

**H6**: Perceived risk will have mediation effect in the influence of SGP on CUV.

### Signal Theory and Perceived Quality

Signaling theory is inspired by information economics, which is used to study the transfer of information between buyers and sellers in the market under the background of information asymmetry (Spence, 1974). For a systematic introduction to the theory, the textbook, “The Theory of Industrial Organization” published by MIT, can be referred (Tirole and Jean, 1988). This study only considers the situation where the seller delivers information to the buyer through the service guarantees. For a commodity, information about its quality before it is sold must be asymmetric between buyers and sellers, especially for commodities with high technical content or extremely similar appearances, such as automobiles, various electronic products, etc. Even after the sale, many attributes of quality, such as durability, safety, etc., cannot be accurately judged by the buyer in a short period of time. As a tool for high-quality product suppliers, service guarantees can effectively convey the information of their own product quality to consumers. On the other hand, for the inferior product suppliers, due to their own product quality standards, once they use a service guarantee equivalent to that of high-quality suppliers would face huge compensation costs (Ippolito, 1990). Consumers can use this logic to distinguish the quality of the product of the seller. When purchasing a product with a service guarantee, it is implied by the information carried by it. The customers would think that the quality of the product is excellent (Grossman, 1981). Of course, this phenomenon can also be used by sellers to mislead consumers. Therefore, the binding force of service guarantees and interpretation of service guarantees by consumers are also the factors that need to be referred to. However, this would be the same as the PSA and service guarantees perception in this study. In short, consumers will still accept the signals sent by sellers in this form (Boulding and Kirmani, 1993), and this has become one of the main means for merchants to build consumer confidence (Lee and Khan, 2012). Therefore, the following hypotheses are proposed:

**H7**: Service guarantee perception will positively affect perceived quality (PEQ) significantly.

**H8**: Perceived quality will positively affect CUV.

**H9**: Perceived quality will have mediation effect in the influence of SGP on CUV.

**FIGURE 1:**
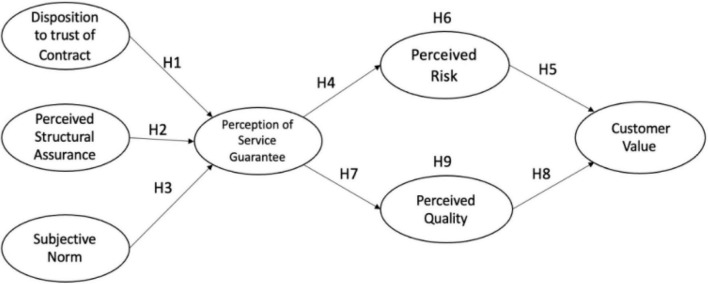
Research model.

In short, this research developed the related concepts of service guarantee and studies its influence on enterprises and consumers in the Chinese context. This study took disposition to trust of contract, PSA, and SUN as the independent variables of SGP. PER and PEQ were the mediating variables between service guarantee and CUV. CUV was the dependent variable. After the literature review, the research model is demonstrated in Figure 1.

## Materials and Methods

### Data Collection

Since the core construct of this study, ‘‘service guarantee perception’’ is the subjective judgment of consumers, the population which the sample is supposed to represent is the whole population of China without any preference. Therefore, this study used a questionnaire survey to collect sample data through the largest online questionnaire platform in China, ‘‘Questionnaire Star’’^1^, along with the social network of the researcher. The research target is the residents in China. Based on the calculation results of the sample size calculator from the survey system website^2^, under 95% of confidence level and 5% of the confidence interval (CI), the sample size should be larger than 384. During the period from 2021/2/1 to 2021/3/31, a total of 696 questionnaires were returned. After the validity review, 574 valid questionnaires were counted.

### Measurement Instrument

The design of the questionnaire includes two parts: basic consumer information and consumer perception. The basic information includes four individual characteristics: gender, age, income, and education level, which are four objective characteristics that are most likely to affect the disposition to trust of contract, PSA, and SUN in H1, H2, and H3. The rest of the questionnaire is developed to measure the 7 latent variables in the above research model, using Likert’s 7-point scale from strongly disagree (1) to strongly agree (7) (Appendix). Using this method, the measurement includes the disposition to trust of contract, PSA, SUN, SGP, PER, PEQ, and CUV of the subject.

Regarding the measurement of disposition to trust of contract, this study compared Lowry et al. (2014) and the research from Wang et al. (2015). In the study of integrity, competence, and trusting stance, seven questions were raised in four subconstructs. The measurement of SUN contained six items, which came from the study of Taufique and Vaithianathan (2018). The measurement of PSA came from the Gefen et al. (2003) research on online shopping and technology acceptance model (TAM) theory. Among them, the measurement logic of structural assurance was highly consistent with the needs of this research. The measurement of SGP came from a 2017 study by Boccia and Sarnacchiaro (2018). Then, PER is a very commonly used variable. This study adopted the measurement of Stone and Grønhaug, 1993, which laid the foundation for the quantification of this variable. For PEQ, this study used the measurement by the search of Yoo and Donthu (2000) on brand added value in 2,000 because their connection of PEQ and perceived value is almost the same with this study. Since the data collection for this study was conducted on general Chinese consumers, the measurement instruments were translated from English to Chinese with the help of a professional English translator. To ensure the accuracy of the translation results, two graduate students in English major helped to back-translate the Chinese questionnaire to English and proofread it with the initial English measurement instrument.

### Data Analysis

This research is essentially a confirmatory study of the relationship between variables. Coupled with the continuity of the quantification method of the Likert scale, the structural equation model (SEM) would be a very reasonable choice. However, due to the comprehensive consideration of the influencing factors of the core variable, SGP, the four category variables of gender, age, education level, and income were also included in the questionnaire. The discreteness of the above four categorical variables determines that they cannot be quantified using the Likert scale and are not suitable for SEMs. Therefore, the first part of the data analysis of this study is the descriptive statistics of categorical variables under SPSS, including frequency, standard deviation, and other statistical methods. At the same time, the description statistics would include the mean value of continuous variables as well. Later, to ensure that the model can be effectively applied to the SEM, refer to the research of Anderson and Gerbing (1998). Moreover, this research continues to analyze the convergence validity, discrimination validity, and model fitness of continuous variables. On the premise of passing the above tests, the model was finally analyzed by structural equation modeling using AMOS, including path analysis between variables and analysis of the mediation effect of specific variables.

## Results

### Descriptive Statistical Analysis

#### Frequency Distribution

In this study, demographic data such as gender, age, education level, and income was investigated shown as Table 1. In gender, female account for the largest number (359, 62.54%). In age, under 25 account for the largest number (282, 49.13%). In education level, undergraduate account for the largest number (322, 56.1%). In income, under 4,000 account for the largest number (345, 60.1%).

**TABLE 1:** Frequency distribution.

Variable	Value label	Value	Frequency	Valid percent	Cum percent
Gender	Male	1	215	37.46	37.46
	Female	2	359	62.54	100.00
		Total	574	100.0	
Age	Under 25	1	282	49.13	49.13
	26–35	2	98	17.07	66.20
	36–45	3	71	12.37	78.57
	45–55	4	54	9.41	87.98
	55–65	5	34	5.92	93.90
	Over 65	6	35	6.10	100.00
		Total	574	100.0	
Education level	Below junior high school	1	46	8.01	8.01
	High school/technical secondary school and junior college	2	95	16.55	24.56
	Undergraduate	3	322	56.10	80.66
	Master degree and above	4	111	19.34	100.00
		Total	574	100.0	
Income	Under 4,000	1	345	60.10	60.10
	4,000–8,000	2	134	23.34	83.45
	8,000–15,000	3	62	10.80	94.25
	Over 15,000	4	33	5.75	100.00
		Total	574	100.0	

#### Item Statistical Analysis

The average values are all between 2.39 and 6.06. The standard deviations of all questions are between 1.04 and 1.51, showing the consistency of each question that the participates responded. The skewness ranged from −1.64 to 1.27, and the kurtosis valued from 0.01 to 4.02, which are consist with the suggestions that the absolute value of skewness is less than 2 and the absolute value of kurtosis is less than 7 (Kline, 2011). Thus, the data is normally distributed as shown in Table 2.

**TABLE 2:** Item statistical analysis.

Variable	*N*	Mean	Std Dev	Kurtosis	Skewness
DTC1	574	6.06	1.04	3.11	−1.56
DTC2	574	5.40	1.38	0.10	−0.83
DTC3	574	5.67	1.06	0.58	−0.78
DTC4	574	5.80	1.12	2.30	−1.33
DTC5	574	5.60	1.21	0.96	−1.03
DTC6	574	5.55	1.25	1.02	−1.08
DTC7	574	5.24	1.44	0.09	−0.81
SUN1	574	5.35	1.40	0.61	−1.03
SUN2	574	5.78	1.14	3.19	−1.46
SUN3	574	5.80	1.15	3.28	−1.51
SUN4	574	5.52	1.24	1.68	−1.21
SUN5	574	5.76	1.13	4.02	−1.64
SUN6	574	5.53	1.19	2.04	−1.18
PSA1	574	5.38	1.41	0.91	−1.1
PSA2	574	5.26	1.43	0.67	−1.00
PSA3	574	5.31	1.38	1.01	−1.06
PSA4	574	5.32	1.34	1.02	−1.03
PSA5	574	5.57	1.15	1.57	−1.06
PSA6	574	5.31	1.36	1.15	−1.09
SGP1	574	5.27	1.26	1.33	−1.06
SGP2	574	5.67	1.27	1.86	−1.27
SGP3	574	5.30	1.46	0.29	−0.91
SGP4	574	5.63	1.27	1.86	−1.32
SGP5	574	5.30	1.32	0.64	−0.87
SGP6	574	5.66	1.15	1.85	−1.22
PER1	574	2.39	1.20	1.82	1.24
PER2	574	2.75	1.43	0.41	0.94
PER3	574	2.79	1.39	0.36	0.88
PER4	574	2.43	1.22	1.79	1.27
PER5	574	2.48	1.22	1.59	1.17
PER6	574	2.48	1.25	1.53	1.20
PEQ1	574	5.34	1.23	0.74	−0.84
PEQ2	574	5.36	1.23	0.77	−0.86
PEQ3	574	5.31	1.21	0.44	−0.78
PEQ4	574	5.36	1.21	0.81	−0.85
PEQ5	574	5.24	1.28	0.72	−0.82
CUV1	574	5.18	1.29	0.04	−0.66
CUV2	574	5.45	1.22	1.07	−1.01
CUV3	574	5.37	1.25	0.69	−0.87
CUV4	574	5.30	1.25	0.32	−0.73
CUV5	574	5.56	1.16	1.80	−1.15
CUV6	574	5.51	1.14	1.30	−0.97
CUV7	574	5.54	1.2	1.37	−1.09
CUV8	574	5.13	1.51	0.01	−0.83

*DTC, Disposition to Trust of Contract; SUN, Subjective Norms; PSA, Perceived Structural Assurance; SGP, Service Guarantee Perception; PER, Perceived Risk; PEQ, Perceived Quality; and CUV, Customer Value.*

### Measurement Model Verification

#### Convergent Validity

An intact SEM analysis should be divided into at least two stages according to previous suggestion (Anderson and Gerbing, 1998). The first stage includes the Measurement Model, and the second stage was the assessment of the structural model. Confirmatory Factor Analysis (CFA), which was equal to the measurement model assessment was a part of the SEM. In this research, the assessment and reduction of CFA measurement model variables are corrected based on the two-step model proposed by Kline (2011). The intact SEM model report can be carried out only if the degree of fitting of the measurement model was acceptable.

The maximum likelihood method was adopted for the measurement model. The parameters for estimation include factor loading, reliability, convergent validity, and discrimination validity. Non-standardized factor loading, standard error, significance testing, standardized factor loading, multiple correlation square, composite reliability, and average variance extracted are provided in Table 3. According to the standard proposed by Fornell and Larcker (1981) about the convergent validity:

**TABLE 3:** Results for the convergent validity.

Construct	Item	Item reliability		Construct reliability	Convergence validity
		Std.	SMC	CR	AVE
DTC	DTC1	0.638	0.407	0.877	0.506
	DTC2	0.642	0.412		
	DTC3	0.773	0.598		
	DTC4	0.760	0.578		
	DTC5	0.745	0.555		
	DTC6	0.740	0.548		
	DTC7	0.668	0.446		
SUN	SUN1	0.762	0.581	0.896	0.589
	SUN2	0.789	0.623		
	SUN3	0.752	0.566		
	SUN4	0.798	0.637		
	SUN5	0.708	0.501		
	SUN6	0.793	0.629		
PSA	PSA1	0.854	0.729	0.910	0.632
	PSA2	0.894	0.799		
	PSA3	0.884	0.781		
	PSA4	0.760	0.578		
	PSA5	0.708	0.501		
	PSA6	0.635	0.403		
SGP	SGP1	0.768	0.590	0.896	0.590
	SGP2	0.784	0.615		
	SGP3	0.783	0.613		
	SGP4	0.760	0.578		
	SGP5	0.829	0.687		
	SGP6	0.678	0.460		
PER	PER1	0.599	0.359	0.875	0.541
	PER2	0.767	0.588		
	PER3	0.773	0.598		
	PER4	0.797	0.635		
	PER5	0.745	0.555		
	PER6	0.713	0.508		
PEQ	PEQ1	0.788	0.621	0.901	0.646
	PEQ2	0.841	0.707		
	PEQ3	0.816	0.666		
	PEQ4	0.835	0.697		
	PEQ5	0.734	0.539		
CUV	CUV1	0.700	0.490	0.914	0.572
	CUV2	0.730	0.533		
	CUV3	0.809	0.654		
	CUV4	0.837	0.701		
	CUV5	0.802	0.643		
	CUV6	0.789	0.623		
	CUV7	0.759	0.576		
	CUV8	0.597	0.356		

*DTC, Disposition to Trust of Contract; SUN, Subjective Norms; PSA, Perceived Structural Assurance; SGP, Service Guarantee Perception; PER, Perceived Risk; PEQ, Perceived Quality; and CUV, Customer Value.*

(1) Standardized Factor Loading of each target variable was higher than 0.50;
(2) Composite Reliability was higher than 0.60;
(3) Average variance extracted was higher than 0.50.

Therefore, as shown in Table 3, all standardized factor loadings between 0.597 and 0.894 are regarded as reasonable to show that all titles have title reliability. The composite reliability of the research dimension was between 0.875 and 0.914. All of them exceed 0.7, which meets the standard suggested by Nunnally and Bernstein (1994). The range of final average variance extracted is “0.506–0.646.” All of them exceed 0.5, which meets the standard proposed by Fornell and Larcker (1981) and Hair et al. (2010) indicates good convergent validity of the dimensions.

#### Discriminant Validity

In this research, a rigorous average variance extracted (AVE) method was adopted for the verification of discriminant validity. Fornell and Larcker (1981) suggested that the discriminant validity should be considered with the relationship between the convergent validity and dimension correlation at the same time. Therefore, the AVE square root of each dimension was suggested to be greater than the correlation coefficient between dimensions. If this requirement was met, it indicates that this research model possesses discriminant validity.

As shown in Table 4, in this research, all the AVE root-mean-squares of all dimensions on the diagonal are greater than the correlation coefficient outside the diagonal. Therefore, this research possesses discriminant validity.

**TABLE 4:** Discriminant validity.

	AVE	DTC	SUN	PSA	SGP	PER	PEQ	CUV
DTC	0.506	**0.711**						
SUN	0.589	0.640	**0.767**					
PSA	0.632	0.545	0.695	**0.795**				
SGP	0.590	0.604	0.746	0.724	**0.768**			
PER	0.541	−0.370	−0.456	−0.443	−0.611	**0.736**		
PEQ	0.646	0.382	0.472	0.458	0.633	−0.387	**0.804**	
CUV	0.572	0.355	0.438	0.425	0.587	−0.592	0.713	**0.756**

*DTC, Disposition to Trust of Contract; SUN, Subjective Norms; PSA, Perceived Structural Assurance; SGP, Service Guarantee Perception; PER, Perceived Risk; PEQ, Perceived Quality; and CUV, Customer Value. The items on the diagonal on bold represent the square roots of the AVE.*

### Structural Equation Mode

#### Model Fit

In a previous study, they found in 194 papers of international academic journals, there are nine most commonly reported model fit indices (Jackson et al., 2009). In SEM analysis, if the sample size is larger than 200, it will cause chi-square to inflate leading to decreased model fit (Bollen and Stine, 1992). This study used Bollen–Stine Bootstrap to corrected SEM chi-square. After Bollen–Stine bootstrapping correction, the model fits indices fit all the criteria of suggestions as Table 5 shown.

**TABLE 5:** Model fit.

Model fit	Criteria	Model fit of research model
Chi-square		1433.221
Degree of freedom		850.000
CFI	>0.90	0.971
RMSEA	<0.08	0.033
TLI	>0.90	0.970
GFI	>0.90	0.934
NFI	>0.90	0.934
χ^2^/*df*	<3	1.716
AGFI	>0.80	0.926

*CFI, Comparative Fit Index; RMSEA, Root Mean Square Error of Approximation; TLI, Tucker-Lewis Index; GFI, Goodness of Fit Index; NFI, Normed-Fit Index; χ^2^, Chi-square; df, Degree of Freedom; and AGFI, Adjusted Goodness of Fit Index.*

#### Path Analysis

**FIGURE 2:**
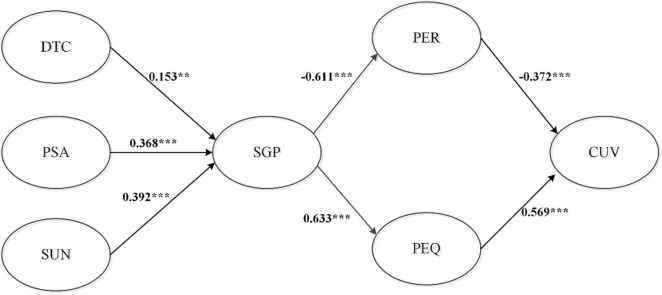
SEM statistic model. Note: *p-value < 0.05; **p-value < 0.01; and ***p-value < 0.001.

Table 6 shows the results of path coefficients. Disposition to trust of contract (b = 0.223, p < 0.05), SUN (b = 0.356, p < 0.05), and PSA (b = 0.295, p < 0.05) significantly influence to SGP. SGP of b = −0.456, p < 0.05 significantly influence to PER. SGP of b = 0.631, p < 0.05 significantly influence to PEQ. PER (b = −0.456, p < 0.001) and PEQ (b = 0.523, p < 0.05) significantly influence to CUV. The results support the research question regarding the validity of the research model. 65.1% of SGP can be explained by disposition to trust of contract, SUN, and PSA constructs. 37.4% of PER can be explained by SGP constructs. 40% of PEQ can be explained by SGP constructs. 62.5% of customer can be explained by PER and PEQ constructs. Figure 2 displays the results of SEM statistic model analysis.

**TABLE 6:** Regression coefficient.

DV	IV	Unstd.	S.E.	Unstd./S.E.	*p*-value	Std.	*R* ^2^
SGP	DTC	0.223	0.065	3.425	0.001	0.153	0.651
	SUN	0.356	0.051	6.959	0.000	0.392	
	PSA	0.295	0.039	7.534	0.000	0.368	
PER	SGP	−0.456	0.043	−10.672	0.000	−0.611	0.374
PEQ	SGP	0.631	0.048	13.161	0.000	0.633	0.400
CUV	PER	−0.456	0.056	−8.159	0.000	−0.372	0.625
	PEQ	0.523	0.044	11.756	0.000	0.569	

*Unstd., Unstandardized regression weight; S.E., Standard Error; Std., Standardized path coefficient; DV, Dependent Variable; IV, Independent Variable; DTC, Disposition to Trust of Contract; SUN, Subjective Norms; PSA, Perceived Structural Assurance; SGP, Service Guarantee Perception; PER, Perceived Risk; PEQ, Perceived Quality; and CUV, Customer Value.*

#### Mediation Effects

As shown in Table 7, the total effect SGP→CUV, *p <* 0.05, bias-corrected CI does not include 0 [CI of SGP→CUV = (0.415.652)]. The existence of total effect was supported. The indirect effect SGP→PER→CUV, *p <* 0.05, bias-corrected CI does not include 0 [CI of SGP→PER→CUV = (0.102.365)]. Thus, the hypothesis that the existence of indirect effect was supported. The indirect effect SGP→PEQ→CUV, *p <* 0.05, bias-corrected CI does not include 0 [CI of SGP→PEQ→CUV = (0.207.495)]. Thus, the hypothesis that the existence of indirect effect was supported.

**TABLE 7:** The analysis of indirect effects.

Effect	Point estimate				Bootstrap 1,000 times	
6-7		**product of coefficients**			**Bias-corrected 95%**	
		**S.E.**	***Z*-Value**	***p*-Value**	**Lower bound**	**Upper bound**

**Total effect**						
SGP→CUV	0.538	0.062	8.625	0.000	0.415	0.652
**Specific indirect effect**						
SGP→PER→CUV	0.208	0.064	3.250	0.001	0.102	0.365
SGP→PEQ→CUV	0.330	0.075	4.377	0.000	0.207	0.495

*DTC, Disposition to Trust of Contract; SUN, Subjective Norms; PSA, Perceived Structural Assurance; SGP, Service Guarantee Perception; PER, Perceived Risk; PEQ, Perceived Quality; and CUV, Customer Value.*

#### Differentiation Analysis of Categorical Variables

##### Gender

In SUN, PSA, SGP, PEQ, CUV variables, the means of the male are greater than that of the female. And in disposition to trust of contact (DTC), PER variables, the means of the male was less than those of the female. Except for the SGP, PEQ, and CUV in the male was significantly higher than that in the female (*p <* 0.05). Other construct has no significant difference between the male and the female. The results were shown in Table 8.

**TABLE 8:** *t*-test by gender.

	Gender	*N*	Mean	Std. Deviation	Mean difference	S.E. difference	*t*	*df*	*p*
DTC	Male	215	5.61	1.09	−0.02	0.08	−0.19	572.00	0.847
	Female	359	5.62	0.80					
SUN	Male	215	5.71	1.08	0.13	0.08	1.58	572.00	0.114
	Female	359	5.58	0.91					
PSA	Male	215	5.40	1.22	0.07	0.10	0.77	572.00	0.445
	Female	359	5.33	1.05					
SGP	Male	215	5.60	1.16	0.21	0.09	2.34	572.00	0.020
	Female	359	5.39	0.96					
PER	Male	215	2.47	1.09	−0.13	0.09	−1.54	572.00	0.125
	Female	359	2.60	0.95					
PEQ	Male	215	5.51	1.02	0.31	0.09	3.48	572.00	0.001
	Female	359	5.21	1.04					
CUV	Male	215	5.50	1.06	0.19	0.08	2.29	572.00	0.022
	Female	359	5.31	0.93					

*DTC, Disposition to Trust of Contract; SUN, Subjective Norms; PSA, Perceived Structural Assurance; SGP, Service Guarantee Perception; PER, Perceived Risk; PEQ, Perceived Quality; and CUV, Customer Value.*

##### Age

The *F* value of the PSA was significant (*F* = 4.23, *p* = 0.001 < 0.05), indicating at least of one pair of the means in different Age levels in the variable PSA was different. The *post hoc* comparison using Scheffe’s method reveals that the subjects with under 25 have significantly higher 26–35 than that with PSA degrees.

The *F* value of the PER was significant (*F* = 3.14, *p* = 0.008 < 0.05) indicating at least of one pair of the means in different Age levels in the variable PER was different. The *post hoc* comparison using Scheffe’s method reveals that the subjects with 45–55 have significantly higher under 25 than that with PER degrees. The results are shown in Table 9.

**TABLE 9:** Analysis of variance by age.

	Age	*N*	Mean	Std. Deviation	F	*p* Sig.	Scheffe
DTC	Under 25	282	5.64	0.87	0.80	0.549	
	26–35	98	5.47	1.08			
	36–45	71	5.62	0.87			
	45–55	54	5.61	1.06			
	55–65	34	5.77	0.69			
	Over 65	35	5.68	0.86			
	Total	574	5.62	0.92			
SUN	Under 25	282	5.61	0.96	1.14	0.336	
	26–35	98	5.47	1.14			
	36–45	71	5.83	0.79			
	45–55	54	5.67	0.97			
	55–65	34	5.63	1.12			
	Over 65	35	5.69	0.89			
	Total	574	5.63	0.98			
PSA	Under 25	282	5.53	1.04	4.23	0.001	1 > 2
	26–35	98	5.02	1.30			
	36–45	71	5.40	1.07			
	45–55	54	5.37	1.10			
	55–65	34	5.09	1.09			
	Over 65	35	5.05	1.06			
	Total	574	5.36	1.12			
SGP	Under 25	282	5.48	1.09	0.63	0.674	
	26–35	98	5.34	1.07			
	36–45	71	5.54	1.04			
	45–55	54	5.56	0.98			
	55–65	34	5.36	0.90			
	Over 65	35	5.60	0.88			
	Total	574	5.47	1.05			
PER	Under 25	282	2.49	1.03	3.14	0.008	4 > 1
	26–35	98	2.50	0.89			
	36–45	71	2.41	0.93			
	45–55	54	3.00	1.08			
	55–65	34	2.80	1.08			
	Over 65	35	2.55	0.91			
	Total	574	2.55	1.01			
PEQ	Under 25	282	5.42	1.00	1.48	0.196	
	26–35	98	5.20	1.11			
	36–45	71	5.37	1.09			
	45–55	54	5.10	1.06			
	55–65	34	5.26	0.99			
	Over 65	35	5.17	1.02			
	Total	574	5.32	1.04			
CUV	Under 25	282	5.49	0.97	1.92	0.089	
	26–35	98	5.31	0.99			
	36–45	71	5.37	1.09			
	45–55	54	5.27	0.93			
	55–65	34	5.05	0.91			
	Over 65	35	5.23	0.91			
	Total	574	5.38	0.98			

*1, under 25; 2, 26–35; 4, 45–55; DTC, Disposition to Trust of Contract; SUN, Subjective Norms; PSA, Perceived Structural Assurance; SGP, Service Guarantee Perception; PER, Perceived Risk; PEQ, Perceived Quality; and CUV, Customer Value.*

##### Education level

The *F* value of the DTC was significant (*F* = 6.19, *p* = 0 < 0.05) indicating at least of one pair of the means in different Education level levels in the variable DTC was different. The *post hoc* comparison using Scheffe’s method reveals that the subjects with High school/technical secondary school and junior college have significantly higher Undergraduate than that with DTC degrees. The subjects with High school/technical secondary school and junior college have significantly higher Master’s degree and above than that with DTC degrees.

The *F* value of the PSA was significant (*F* = 7.01, *p* = 0.000 < 0.05) indicating at least of one pair of the means in different Education level levels in the variable PSA was different. The *post hoc* comparison using Scheffe’s method reveals that the subjects with High school/technical secondary school and junior college have significantly higher Undergraduate than that with PSA degrees. The subjects with High school/technical secondary school and junior college have significantly higher Master degree and above than that with PSA degrees.

The *F* value of the SGP was significant (*F* = 2.91, *p* = 0.034 < 0.05) indicating at least of one pair of the means in different Education level levels in the variable SGP was different. After Scheffe’s *post hoc* comparison, there was no difference between groups. The results were displayed in Table 10.

**TABLE 10:** Analysis of variance by education level.

	Education level	*N*	Mean	Std. Deviation	*F*	*p*-value	Scheffe
DTC	Below junior high school	46	5.62	1.02	6.19	0.000	2 > 3 2 > 4
	High school/technical secondary school and junior college	95	5.94	0.89			
	Undergraduate	322	5.59	0.82			
	Master degree and above	111	5.40	1.10			
	Total	574	5.62	0.92			
SUN	Below junior high school	46	5.73	0.95	2.12	0.097	
	High school/technical secondary school and junior college	95	5.84	1.11			
	Undergraduate	322	5.57	0.89			
	Master degree and above	111	5.56	1.10			
	Total	574	5.63	0.98			
PSA	Below junior high school	46	5.54	1.10	7.01	0.000	2 > 3 2 > 4
	High school/technical secondary school and junior college	95	5.72	1.01			
	Undergraduate	322	5.33	1.05			
	Master degree and above	111	5.04	1.31			
	Total	574	5.36	1.12			
SGP	Below junior high school	46	5.57	1.08	2.91	0.034	No difference between groups
	High school/technical secondary school and junior college	95	5.74	1.04			
	Undergraduate	322	5.41	1.00			
	Master degree and above	111	5.39	1.14			
	Total	574	5.47	1.05			
PER	Below junior high school	46	2.58	1.15	1.66	0.174	
	High school/technical secondary school and junior college	95	2.37	1.06			
	Undergraduate	322	2.56	0.95			
	Master degree and above	111	2.68	1.04			
	Total	574	2.55	1.01			
PEQ	Below junior high school	46	5.42	1.36	0.60	0.615	
	High school/technical secondary school and junior college	95	5.32	1.24			
	Undergraduate	322	5.28	0.97			
	Master degree and above	111	5.41	0.89			
	Total	574	5.32	1.04			
CUV	Below junior high school	46	5.38	1.27	0.70	0.554	
	High school/technical secondary school and junior college	95	5.51	1.03			
	Undergraduate	322	5.37	0.91			
	Master degree and above	111	5.32	0.99			
	Total	574	5.38	0.98			

*2, High school/technical secondary school and junior college; 3, Undergraduate; 4, Master degree and above; DTC, Disposition to Trust of Contract; SUN, Subjective Norms; PSA, Perceived Structural Assurance; SGP, Service Guarantee Perception; PER, Perceived Risk; PEQ, Perceived Quality; and CUV, Customer Value.*

##### Income

The *F* value of the PSA was significant (*F* = 5.82, *p* = 0.001 < 0.05) indicating at least of one pair of the means in different Income levels in the variable PSA was different. The *post hoc* comparison using Scheffe’s method reveals that the subjects with under 4,000 have significantly higher (4,000–8,000) than that with PSA degrees.

The *F* value of the PER was significant (*F* = 3.65, *p* = 0.013 < 0.05) indicating at least of one pair of the means in different Income levels in the variable PER was different. After Scheffe’s *post hoc* comparison, there was no difference between groups.

The *F* value of the PEQ was significant (*F* = 2.94, *p* = 0.032 < 0.05) indicating at least of one pair of the means in different Income levels in the variable PEQ was different. After Scheffe’s *post hoc* comparison, there was no difference between groups.

The *F* value of the CUV was significant (*F* = 4.09, *p* = 0.007 < 0.05) indicating at least of one pair of the means in different Income levels in the variable CUV was different. The *post hoc* comparison using Scheffe’s method reveals that the subjects with under 4,000 have significantly higher (4,000–8,000) than that with CUV degrees. The analysis results were shown in Table 11.

**TABLE 11:** Analysis of variance by income.

	Income	*N*	Mean	Std. Deviation	F	*p* Sig.	Scheffe
DTC	Under 4,000	345	5.69	0.92	2.27	0.080	
	4,000–8,000	134	5.47	0.86			
	8,000–15,000	62	5.59	0.84			
	Over 15,000	33	5.45	1.18			
	Total	574	5.62	0.92			
SUN	Under 4,000	345	5.68	1.00	0.85	0.469	
	4,000–8,000	134	5.53	0.87			
	8,000–15,000	62	5.57	0.97			
	Over 15,000	33	5.59	1.16			
	Total	574	5.63	0.98			
PSA	Under 4,000	345	5.51	1.12	5.82	0.001	1>2
	4,000–8,000	134	5.12	1.00			
	8,000–15,000	62	5.12	1.18			
	Over 15,000	33	5.14	1.24			
	Total	574	5.36	1.12			
SGP	Under 4,000	345	5.56	1.00	2.26	0.081	
	4,000–8,000	134	5.37	1.00			
	8,000–15,000	62	5.32	1.09			
	Over 15,000	33	5.23	1.47			
	Total	574	5.47	1.05			
PER	Under 4,000	345	2.44	1.00	3.65	0.013	No difference between groups
	4,000–8,000	134	2.69	0.93			
	8,000–15,000	62	2.80	1.06			
	Over 15,000	33	2.66	1.12			
	Total	574	2.55	1.01			
PEQ	Under 4,000	345	5.42	1.06	2.94	0.032	No difference between groups
	4,000–8,000	134	5.13	0.97			
	8,000–15,000	62	5.29	1.02			
	Over 15,000	33	5.13	1.02			
	Total	574	5.32	1.04			
CUV	Under 4,000	345	5.48	1.01	4.09	0.007	1>2
	4,000–8,000	134	5.15	0.90			
	8,000–15,000	62	5.39	0.90			
	Over 15,000	33	5.22	0.97			
	Total	574	5.38	0.98			

*1, under 4,000; 2, 4,000–8,000; DTC, Disposition to Trust of Contract; SUN, Subjective Norms; PSA, Perceived Structural Assurance; SGP, Service Guarantee Perception; PER, Perceived Risk; PEQ, Perceived Quality; and CUV, Customer Value.*

## Discussion and Conclusion

Based on the observation of the fact that the understanding of consumers of business guarantees in the Chinese market was clearly differentiated, this article was inspired by the theory of rational behavior and the theory of planned behavior and studies the main factor of influence of the SGP of customers in the Chinese context. Based on previous studies, a mechanical model for the formation of customer perception guarantees in Chinese contexts and the subsequent influence on CUV was constructed. Through 574 sample responses, this study tested the reliability and validity of the measurement, the SEM fitness, path analysis, and mediation effects. The nine hypotheses were all supported. The research conclusions lay a theoretical foundation for further research on the formation of customer psychological contracts in the Chinese context. The results would provide references for service providers, especially foreign service providers, a better understanding of customer perception of guarantees in the Chinese context and establish active regulatory interventions mechanism, targeted design, and improvement of service guarantee policies.

### Conclusion

The specific conclusions of this research can be summarized into three aspects:

#### Factors Influencing Guarantee Perception in Chinese Context

This study analyzed and verified the influencing factors of SGP under the Chinese scenario. It can be seen from Table 6 that disposition to trust of contract, SUN, and PSA have a significant influence on SGP (*p*-values are 0.000, 0.000, and.001, respectively), which supports the establishment of H1, H2, and H3 in this article. The path coefficients are, respectively: 0.153, 0.392, and 0.368. From these data, we can draw the following conclusions: firstly, the influence of disposition to trust of contract, SUN, and PSA on SGP are all positive, which was in line with common sense and experience. Secondly, SUN have the greatest influence on the SGP, which echoes the research results of scholars mentioned above, such as Peng and Nisbett (1999) and Liao and Xu (2012), that was, SGP of Chinese consumers was most susceptible to the influence of “exemplary norms.” This result reveals the strength of the “human sentiment” of Chinese society. When faced with choices, the opinions of others, especially those close to them, have a very high weight. The cause of this phenomenon was likely to be the same as that of the family collectivism in China since ancient times. It may have been influenced by the long-lasting “society ruled by man” in history. Thirdly, the influence of PSA on SGP was not the same as the influence of SUN (path coefficients are 0.368 and 0.392). This shows that structural assurance is the basis for the general trust of society, which confirms the idea of McCole et al. (2019). Also, that the understanding and correct PSA was an important prerequisite to the SGP of the customer. This result can laterally reflect the achievements of the modern market economy and the construction of a legalized society of China. That is, social rules such as laws and regulations, corporate rules and regulations, and management procedures have been formed equally to the mindset of the judgment standards of Chinese consumers for thousands of years. Finally, although the disposition of trust to contract has a significant influence on the SGP, its influence was far less than the other two variables. A reasonable estimation for this was that with the development of modern business systems and social credit systems, the disposition of Chinese consumers to trust of contract gradually converge. More and more consumers tend to acquiesce that others are honest, although they will still be affected by the education level as will be mentioned below. The overall disposition to trust of contract has not been able to have a strong influence.

#### Differentiation Analysis of Categorical Variables

The above path analysis reveals the weight of influence between the dimensions. Next, this research studies the characteristics of specific customer groups. The purpose of this analysis was to provide a theoretical basis for the classification of customer groups and make it meaningful for management. This study analyzed the differentiated performance of three types of variables in specific dimensions based on the degree of significance, namely the age, education level, and income level of consumers. This supports the relevant influencing factors of disposition of trust (level of education, employment, and compositions of residential area) by Frederiksen. Through Tables 8–10, the following conclusions can be drawn: First, concerning age, the PSA for people under 25 was significantly higher than that of people aged 25–36. People aged 45–55 have significantly higher PER than those under the age of 25. This pair of conclusions suggests that as age increases, PER of customers will also increase. The main reason might be the decline in the PSA. This logical result suggests that young people have a higher level of trust in laws and regulations than older people. This may be due to the rich life experience of the elders, or the difference in legal knowledge education between the two generations. Table 9 shows that consumers with a bachelor’s degree or below has a significantly higher disposition to trust of contract than consumers with a bachelor’s degree, and their PSA was also significantly higher than those with a graduate degree or above. Therefore, this study has reason to believe that the higher the level of education in the Chinese context, the higher the degree of suspicion of contracts. The low PSA maybe an important reason. Considering that age and education level are generally positively correlated, this study believes that the PSA may not have much to do with the understanding of relevant knowledge. Finally, Table 10 shows that people with an income level of less than 4,000 have a higher perceived level of structural assurance and CUV than those with an income level of 4,000 to 8,000. That is, low-income groups have a higher level of PSA compared to middle and high-income groups. The low-income group was easier to satisfy when faced with the same service.

#### The Influence of Service Guarantee Perception on Customer Value

Finally, this study verifies the influence of SGP on CUV through PER and PEQ. The purpose of this verification was also to increase the contribution of the above conclusions to management practices. According to the data in Table 7, SGP significantly affects CUV by restraining PER and promoting PEQ (*p*-value was far less than 0.05), that is, excellent SGP intervention strategies can effectively improve CUV.

### Implication

Apparently, seeking ways to increase CUV was also an important way for companies to gain a competitive advantage (Morris and Holbrook, 1996). Then, while this research confirms the positive influence of SGP on CUV, it proves that companies should use management and the means of intervention to enable customers to positively perceive the service guarantee of the company. To achieve this goal in the Chinese context, service providers should first focus on the subjective normative effect of society, especially the exemplary normative effect, from a strategic point of view. By strengthening the fulfillment quality of self-guarantee, the overall perception of service assurance among consumers would increase, thereby increasing the demonstration effect on the individual. In addition, if it is possible to identify the group that positively perceives the service guarantee through the management of the customer relationship to accurately push specific products or guarantees, it will also increase the value of the feelings of the group. Last but not the least is to try to improve the perception of structural assurance of the customer. Although the ability of the service provider to intervene in external structural assurances (such as laws and regulations) was generally not high, its own internal rules and regulations still have autonomy. Moreover, the attributes of PSA provide service providers with considerable subjective initiative, such as actively publicizing relevant government laws and regulations, typical cases accepted by relevant departments, and rules and regulations on service guarantees in their own management system, and so on.

Based on the above strategies, service providers should notice a fact that not every product requires a same level of guarantee, a key to a successful design of guarantee is its target user. Therefore, service providers should pay attention to the collection, estimation, and management of customer information at the operational level. The previous research found that at least age, education level, and income level are closely related to the influence of SGP on CUV. Information collection and management of these dimensions can enable managers to pinpoint specific customer preferences and provide customers with more suitable services. Younger people with low education and income levels have a higher perception of structural assurances. This can be explained by their relatively little understanding of the failure of structural assurances or cases. Therefore, for a product aimed to this group of customers, such as a cellphone or PC with a price relatively low, a guarantee could cause a remarkable increase of CUV, and the publicity of structural assurances can focus on consumers outside of this group. At the same time, the rise of age will lead to an increase in PER. Groups with higher education levels are less inclined to believe in contracts. Therefore, in older groups, especially consumers with higher education levels, a product aimed to this group of customers won’t necessarily come with a comprehensive guarantee, and a service provider could promote of the service guarantee through their disposition of trust to a contract. The SGP reduces their PER, thereby increasing their satisfaction.

### Limitation and Future Direction

Like all empirical research, this research has certain limitations. Firstly, the core variable of this research was the SGP, which was highly subjective. Many variables can affect it. The three variables confirmed in this study can explain most of the varieties. It was believed that further research will strengthen the integrity of the variables. Secondly, since this research uses the form of online questionnaire data acquisition, it will inevitably receive the influence of different groups on the acceptance of the Internet. The result was that the proportion of young people in the sample was large. Most of the education level of the samples was a bachelor’s degree or below, and the proportion of people with a monthly income below 4,000 was larger than that of other groups. If follow-up research can improve the data collection method, and then make the sample more evenly distributed among the variables of each category, perhaps more accurate results can be obtained. Finally, the two variables that have the greatest influence on SGP, namely SUN, and PSA, can have further discussion in depth. The influence of SUN in the Chinese context goes far beyond the SGP, it was a very important and complex variable. The research on it can be conducted through the professional knowledge of various disciplines such as management, sociology, behavior, history, culture, economics, and other factors.

## Data Availability Statement

The raw data supporting the conclusions of this article will be made available by the authors, without undue reservation.

## Author Contributions

HY: conceptualization. HY and SZ: formal analysis. HY, SZ, and M-CH: investigation, methodology, writing—original draft, and writing—review and editing. All authors have read and agreed to the published version of the manuscript.

## Conflict of Interest

The authors declare that the research was conducted in the absence of any commercial or financial relationships that could be construed as a potential conflict of interest.

## Publisher’s Note

All claims expressed in this article are solely those of the authors and do not necessarily represent those of their affiliated organizations, or those of the publisher, the editors and the reviewers. Any product that may be evaluated in this article, or claim that may be made by its manufacturer, is not guaranteed or endorsed by the publisher.

## Footnotes

1
https://www.wjx.cn

2
https://www.surveysystem.com/sscalc.htm

## Appendix

### Questionnaire Items

**TABLE A1:**

*Disposition to trust of contract (scaling from “Strongly disagree” to “Strongly agree” on a seven-point scale)*
DTC1 In general, people care a lot about the contracts they make with others.
DTC2 In most cases, people are more inclined to think of others at the same time than to look out for their own interests only
DTC3 people usually keep their promises.
DTC4 In most cases, people will do something about their guarantee.
DTC5 most people treat people honestly
DTC6 I choose to trust others until a good reason appears.
DTC7 I choose to trust a new acquaintance until definite evidence appears.
*Subjective norm (scaling from “Strongly disagree” to “Strongly agree” on a seven-point scale)*
SUN1 Most of my family members believe that merchants keep their service promises (e.g., warranty, no-excuse returns, free lifetime maintenance, etc.).
SUN2 Most of my family members think it is wise to buy items that come with a service promise (e.g., warranty, no-excuse returns, free lifetime maintenance, etc.).
SUN3 Most of my friends around me think that if possible they should buy goods that come with a service promise (such as warranty, no-excuse return, free lifetime maintenance, etc.).
SUN4 Most of my friends around me think that merchants will be responsible for their service promises (such as warranty, no-excuse return, free lifetime maintenance, etc.).
SUN5 Most of the colleagues around me think that one should go for goods with service promises (such as warranty, no-excuse return, free lifetime maintenance, etc.).
SUN6 Most of my colleagues around me believe that merchants are honest about their service promises (e.g., warranty, no-excuse returns, free lifetime maintenance, etc.).
*Perceived structural assurance (scaling from “Strongly disagree” to “Strongly agree” on a seven-point scale)*
PSA1 I feel at ease when I deal with a merchant because I know that the China Consumers’ Association (CC) can protect me.
PSA2 I am not worried about merchants defrauding me because I know that the Civil Code/Consumer Protection Law can protect me.
PSA3 I feel safe when I shop because I know that market regulators (such as the business sector) can protect me.
PSA4 A merchant that provides a 400-customer service number will make me feel more trustworthy.
PSA5 I would feel comfortable with a merchant if he disclosed their dispute handling process and mechanism to me.
PSA6 I would feel comfortable with a merchant if I could find him on a well-known search site (such as Baidu, Google, etc.).
*Service Guarantee Perception (scaling from “Strongly disagree” to “Strongly agree” on a seven-point scale)*
PSG1 I believe in the service promises offered by the merchant (e.g., warranty, no-excuse return, free lifetime maintenance, etc.).
PSG2 It is a serious problem for merchants to breach service promises (such as warranty, no-excuse returns, free lifetime maintenance, etc.).
PSG3 I think merchants offer service promises (such as warranty, no-reason returns, free lifetime maintenance, etc.) from the perspective of the customer’s interest rather than a marketing slogan.
PSG4 Service promises (such as warranty, no-reason returns, free lifetime maintenance, etc.) are important factors that I consider when I consume.
PSG5 Merchants care deeply about their ability to keep their service promises (e.g., warranty, no-excuse returns, free lifetime maintenance, etc.).
PSG6 Even if it’s a little more expensive, I would go for the item that comes with a service promise (such as warranty, no reason to return, free lifetime maintenance, etc.).
*Perceived Risk (scaling from “Strongly disagree” to “Strongly agree” on a seven-point scale)*
PER1 If I buy an item I don’t need, even if it comes with a service guarantee (e.g., warranty, no-questions-asked return, free lifetime maintenance, etc.), I still worry about facing certain losses.
PER2 Even though the product comes with a service guarantee (e.g., warranty, no-excuse return, free lifetime maintenance, etc.), I still worry that the money spent on this purchase is a waste.
PER3 Even though the item comes with a service guarantee (e.g., warranty, no-reason returns, free lifetime maintenance, etc.), I still wonder if I shouldn’t spend my money more elsewhere.
PER4 Even if the product comes with a service guarantee (such as warranty, no reason to return, free lifetime maintenance, etc.), I still worry whether it does what it is advertised to do.
PER5 Even though the item comes with a service guarantee (e.g., warranty, no-excuse return, free lifetime maintenance, etc.), I am still concerned about whether the item will meet my needs.
PER6 Overall, service warranties (e.g., warranties, no-excuse returns, free lifetime maintenance, etc.) do not protect against potential losses.
Perceived Quality (scaling from “Strongly disagree” to “Strongly agree” on a seven-point scale)
PEQ1 Goods that come with a service guarantee (such as warranty, no reason to return, free lifetime maintenance, etc.) are generally of good quality.
PEQ2 Goods that come with a very good service guarantee (e.g., warranty, no-excuse returns, free lifetime maintenance, etc.) are generally of excellent quality.
PEQ3 Goods that come with a service guarantee (such as warranty, no-excuse return, free lifetime maintenance, etc.) generally function well.
PEQ4 Goods that come with a service guarantee (e.g., warranty, no-excuse return, free lifetime maintenance, etc.) are generally reliable and less prone to errors or failures.
PEQ5 Goods with service guarantee (such as warranty, no reason to return, free lifetime maintenance, etc.) are generally advanced in workmanship and excellent in workmanship.
*Customer Value (scaling from “Strongly disagree” to “Strongly agree” on a seven-point scale)*
CUV1 Usually, goods that come with a service guarantee (such as warranty, no-excuse return, free lifetime maintenance, etc.) are generally priced more reasonably.
CUV2 The service guarantee (e.g., warranty, no-excuse return, free lifetime maintenance, etc.) saves me time once problems arise after purchase
CUV3 It is usually a good shopping experience to buy goods that come with service guarantees (such as warranty, no-excuse returns, free lifetime maintenance, etc.).
CUV4 My purchases that come with service guarantees (e.g., warranty, no-questions-asked returns, free lifetime maintenance, etc.) all provide me with a high economic value.
CUV5 The service guarantee that comes with the product (e.g., warranty, no-excuse return, free lifetime maintenance, etc.) can alleviate my concerns.
CUV6 The service guarantee (such as warranty, no-excuse return, free lifetime maintenance, etc.) that comes with the item will make me more satisfied.
CUV7 The service guarantee that comes with the item (e.g., warranty, no-excuse return, free lifetime maintenance, etc.) would increase the likelihood that I would buy it again.
CUV8 I think it is reasonable to pay extra for service guarantees (e.g., warranty, no-excuse returns, free lifetime maintenance, etc.).
